# Atlas – a data warehouse for integrative bioinformatics

**DOI:** 10.1186/1471-2105-6-34

**Published:** 2005-02-21

**Authors:** Sohrab P Shah, Yong Huang, Tao Xu, Macaire MS Yuen, John Ling, BF Francis Ouellette

**Affiliations:** 1 UBC Bioinformatics Centre, University of British Columbia, Vancouver, BC, Canada

## Abstract

**Background:**

We present a biological data warehouse called Atlas that locally stores and integrates biological sequences, molecular interactions, homology information, functional annotations of genes, and biological ontologies. The goal of the system is to provide data, as well as a software infrastructure for bioinformatics research and development.

**Description:**

The Atlas system is based on relational data models that we developed for each of the source data types. Data stored within these relational models are managed through Structured Query Language (SQL) calls that are implemented in a set of Application Programming Interfaces (APIs). The APIs include three languages: C++, Java, and Perl. The methods in these API libraries are used to construct a set of loader applications, which parse and load the source datasets into the Atlas database, and a set of toolbox applications which facilitate data retrieval. Atlas stores and integrates local instances of GenBank, RefSeq, UniProt, Human Protein Reference Database (HPRD), Biomolecular Interaction Network Database (BIND), Database of Interacting Proteins (DIP), Molecular Interactions Database (MINT), IntAct, NCBI Taxonomy, Gene Ontology (GO), Online Mendelian Inheritance in Man (OMIM), LocusLink, Entrez Gene and HomoloGene. The retrieval APIs and toolbox applications are critical components that offer end-users flexible, easy, integrated access to this data. We present use cases that use Atlas to integrate these sources for genome annotation, inference of molecular interactions across species, and gene-disease associations.

**Conclusion:**

The Atlas biological data warehouse serves as data infrastructure for bioinformatics research and development. It forms the backbone of the research activities in our laboratory and facilitates the integration of disparate, heterogeneous biological sources of data enabling new scientific inferences. Atlas achieves integration of diverse data sets at two levels. First, Atlas stores data of similar types using common data models, enforcing the relationships between data types. Second, integration is achieved through a combination of APIs, ontology, and tools. The Atlas software is freely available under the GNU General Public License at:

## Background

One important goal in bioinformatics is to integrate data from disparate sources of heterogeneous biological information. Data integration allows us to assemble targeted data reagents for bioinformatics analyses, and to discover scientific relationships between data. Most public repositories of biological data focus on deriving and providing one particular type of data, be it biological sequences (e.g., GenBank [1], UniProt [2]), molecular interactions (The Biomolecular Interaction Network Database (BIND) [3-5], The Human Protein Reference Database (HPRD) [6]), or gene expression (The Stanford microarray database [7]). Integrating these disparate sources of data enables researchers to discover new associations between the data, or validate existing hypotheses.

Several recent studies have demonstrated the power of integrative bioinformatics. Using data from genomic sequences and annotations, mRNA expression, and subcellular localization, Mootha *et al *were able to use bioinformatics approaches to identify one of the disease genes responsible for Leigh syndrome [8]. In another example of an integrative bioinformatics approach, Stuart *et al *used existing publicly available data to generate hypotheses about the functional roles of gene sets [9]. These two examples illustrate the potential of querying integrated public data to reveal novel relationships.

However, working with publicly available biological data can be challenging due to the volume and complexity of the data types. With the proliferation of massive, publicly available data sets, researchers need a way to readily access this data. Querying distributed data has inherent limitations such as the server resource restrictions of the remote resource, concerns of secure data transmission over the internet, and of course the actual logistics of querying distributed resources. In such an environment, the distributed search space is difficult to process in a high-throughput way, and requires complex queries to tie together the heterogeneous data. Consequently, there is a need for a data integration solution that facilitates search and retrieval in an efficient, flexible, high-throughput manner.

Several active solutions are available that attempt to integrate data and that provide the tools to retrieve that data. We have grouped these existing systems into three major categories, based on how the data is stored and integrated: full record, SQL-based, and distributed.

Full record systems like SRS [10] and Entrez [11] store the intact record in a table and extract specific fields to index and cross-reference. SeqHound [12] is a powerful system that stores Entrez information (fully annotated sequence and structure information) locally and can be accessed programmatically through application programming interfaces APIs. Much like Entrez and SRS, fully intact records are stored in SeqHound, with specific fields indexed. The major advantages of SeqHound over Entrez is that it is locally installable and provides API access to the data. SeqHound highlights the power and utility of a locally installable warehouse.

SQL-based systems implement relational models to store data. This allows SQL-level access to specific parts of the data model, enabling detailed queries on the data for greater specificity of results. The data in relational models are stored as primitive data types as opposed to storing fully intact records that need parsing or processing to access the parts therein. For example, sequences and their annotated biological features can be stored in their own fields in the database, permitting 'substring' operations to extract parts of the sequence that span a particular feature type using SQL. Systems like EnsMart [13] and DBGET/LinkDB [14] provide data in a relational form, such that the power of SQL is at users' disposal. EnsMart's relational back-end provides users with the ability to construct intricate queries on the data by taking advantage of SQL.

Distributed systems implement software to access heterogeneous databases that are dispersed over the internet. JXP4BIGI [15] have created a generalized method to access, extract, transform, and integrate distributed data. The tool acts as a middle-ware for constructing a local instance of a data warehouse. This system is customizable, versatile and uses industry standard data modeling, distribution, and presentation software. BioMOBY [16] is a semantic-based system utilizing ontologies, and a services model to support user queries. TAMBIS [17], like BioMOBY, is also a semantic-based system, and is also service-model driven. These semantic web implementations do not house the data locally, but rather query the original data provider for available services before sending queries to that particular data provider. These systems are quite powerful for interrogating disparate data sources of information. However, a disadvantage is that large queries may take a long time to return or may not be returned at all due to server resource restrictions. As well, the level of data integration is only at the services level, rather than at a field-based level which can provide much better resolution for queries.

Atlas is a versatile, flexible, and extensible data warehouse that provides a solution to these challenges. Our approach establishes common relational data models enabling the reuse of each class of data model to store all data of the same type. For example, a single interaction data model is used to store information from any of the interaction data sets such as BIND, MINT, EBI IntAct [18], Database of Interacting Proteins (DIP) [19], and HPRD.

Instances of these data models, once populated by the source data, can then be interrogated using the developed retrieval APIs. These APIs encapsulate the SQL calls used for fine granular access to the data. Furthermore, ontological information stored in these databases captures the relationships between the many data types. Finally, tools are developed that capitalize on the API methods, to facilitate application specific demands of end-users, ranging from simple queries of specific data types, to complex queries that infer molecular interactions across species. Atlas then, is designed for use by a wide audience from biologist to software developer.

## Construction and content

**Figure 1:**
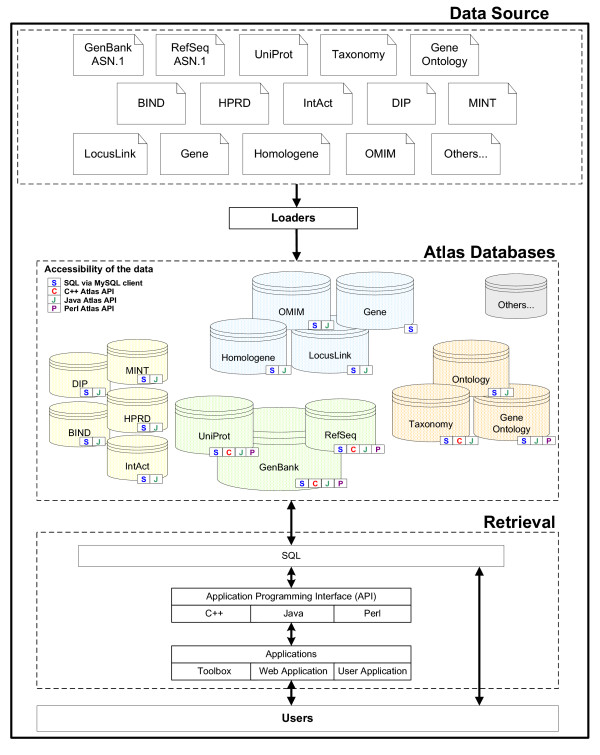
**Architecture of the Atlas data warehouse**. The data integrated in Atlas are first downloaded as data files from the public repositories shown in the **Data Source **panel. These data files are then parsed and loaded into the MySQL relational databases using the Atlas loaders. The **Atlas Databases **panel shows the databases grouped by biological theme. These groups are sequences (green), molecular interactions (yellow); genes and functional categorization (blue); and ontologies (orange). For each database the available data retrieval methods are marked as SQL (**S**), C++ Atlas API (**C**), Java Atlas API (**J**), and Perl Atlas API (**P**). The **Retrieval **panel shows the flexible, layered architecture of the interfaces to the databases. Data can be accessed directly using the MySQL client with SQL statements, through the APIs in C++, Java, and Perl, and through the end-user applications implemented in the Toolbox. The APIs can also be used to implement web-based tools, or standalone applications.

The Atlas system is made up of five main parts: 1) the source data, 2) the ontology system, 3) the relational data models, 4) the APIs, and 5) the applications (see Figure 1). The following sections outline the Atlas architecture in detail.

### Source data

We categorize the Atlas data sources into four main groups: 'sequence', 'molecular interactions', 'gene related resources', and 'ontology' (Figure 1). Currently, the data sources that fall into these categories are: 'sequence', GenBank, RefSeq [11], and UniProt ; 'molecular interactions', HPRD, BIND, DIP, IntAct, and MINT; 'gene related resources', Online Mendelian Inheritance in Man (OMIM) [20], LocusLink [11,21], Entrez Gene [22], and HomoloGene [11,23]; and 'ontology', NCBI Taxonomy [11,24], and Gene Ontology [25,26]. Table 1 lists each of the sources of data incorporated into Atlas, and provides URLs where those sources can be found. Note that GenBank refers to the integrated records from the International Nucleotide Sequence Database Collaboration (GenBank [11], DDBJ [27], and EMBL [28]).

### Relational data models (schema design)

This section describes the composition of the data models of the source data included in Atlas. The data models we present here are implemented in MySQL [29], an open source relational database management system (RDBMS). As such we only provide Data Definition Language (DDL) files that are compatible with MySQL. Currently there are no plans to port these to other RDBMS systems.

#### Ontology

Ontologies serve to define the concepts and relationships both within a system and between systems. This vocabulary of concepts and relationships is representative of a given expert domain of discourse such as sequences, gene annotations, and taxonomy. In Atlas, ontologies are categorized into two classes: Atlas defined ontologies and external ontologies. The Atlas defined ontologies are used to represent the concepts and relationships found specifically within Atlas, as well as to characterize concepts and relationships implicitly defined by the GenBank Sequence Feature data model. External ontologies include such things as NCBI Taxonomy for organism classification, Gene Ontology for gene annotations enabling categorization of biological features based on function, process, and cellular component, and the Proteomics Standards Initiative Molecular Interaction Standard (PSI-MI) controlled vocabulary [30]. The Atlas internal ontologies contain definitions of terms such as identifier types like accession numbers, GI numbers, PSI-MI terms and identifiers, PubMed identifiers, file format types like XML, relationship terms, and concepts like GenBank Sequence Features and Feature Qualifiers, Sequencing Techniques. This part of the Atlas ontology consists of three tables: **Ontology **which include terms and definitions, **Ontology_type **that defines ontology source and category, and **Ontology_Ontology **which stores term-term relationships. Foreign key constraints are used to ensure data integrity. In contrast to these tightly integrated ontologies, two other external vocabularies are instantiated as independent MySQL databases: GO and NCBI Taxonomy. These ontologies, unlike the others, do not implement foreign key enforcements to the other database modules. As a result, when ontology terms are updated, references to deleted terms deemed to be invalid are kept in the system until such time a full data set reload is performed.

The Atlas internal ontology exists largely to help describe Sequence Features as they exists in the GenBank Sequence Feature model, as this is the primary data source for features. Neither the Open Biological Ontologies (OBO) [31] relationship terms, nor the Sequence Ontology (SO) [32] relationship terms suited our needs as a feature ontology. We utilize the basic relationships similarly found in OBO and SO, such as 'is-a', 'part-of', and 'inverse-of' but we also define more specific terms such as 'is-synonym-of', 'refers to PubMed', 'feature-includes-qualifier', and 'gene-contains-promoter'. By defining these specific relationships, we simplify the ontology tree into a flatter structure that is simple to query. In addition, subject-predicate-object triples are not explicitly defined in the internal ontology, but rather are assigned at loading-time as the GenBank Sequence Feature data is parsed and stored into the database. The relationship terms are not necessarily complete, but sufficient for our needs, and as new relationships are encountered, these are added accordingly. For example, we mapped all 66 GenBank feature keys to an entry in our Ontology table, which has enabled us to do feature-level queries for any type of feature in GenBank, or genomes we annotated in-house. We caution the reader that it is generally understood that not all GenBank features have the same informational value, nor quality of information. However, to capture the maximum amount of information, we chose to extract and store all annotated features. With the locations of the features stored in Atlas, sub-sequences of features can be extracted in a high-throughput manner using SQL, the APIs, or the toolbox applications. This is particularly useful, for example, in extracting features like non-coding RNAs from complete genomes, or regions spanning a particular gene of interest. We are actively integrating selected external ontologies, and expanding our internal ontologies. Plans for ontology integration include the National Library of Medicine (NLM) MeSH term and the Microarray Gene Expression Data (MGED) ontology [33]. We are evaluating the option of adopting frame-based ontology representations, and existing ontologies such as TAMBIS Ontology (TaO) [17,34]. In the near future, we will release the Atlas ontology in other formats such as GO flat file, RDF/XML, and OWL. A complete list of the ontologies is available on the Atlas website, and we provide the MySQL dumps for these: .

#### Sequence model

**Figure 2:**
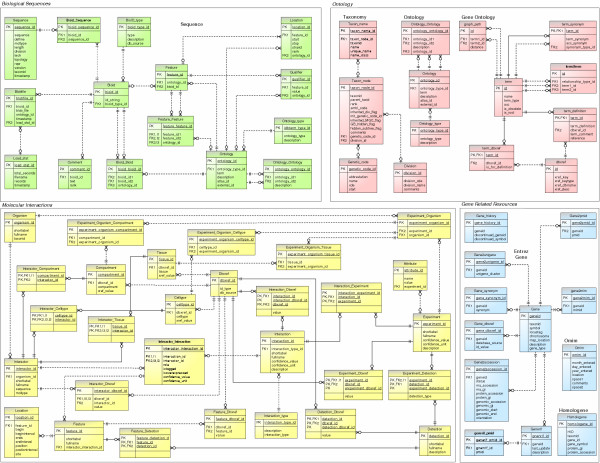
**Atlas database schema**. There are four major functional groups. Biological Sequences: includes instances of GenBank sequences, RefSeq sequences, and UniProt sequences; Molecular Interactions: includes instances of BIND, HPRD, DIP, IntAct and MINT; Gene Related Resources: includes instances of OMIM, Entrez Gene, and LocusLink, and HomoloGene; and Ontology: includes instances of Taxonomy, Atlas internal ontologies, Gene Ontology, and PSI-MI ontologies.

The schema for sequences is organized into three main parts: *sequence*, which stores the sequence string and associated meta-data such as sequencing technique and molecule type; sequence *identifiers*, for which all identifiers that appear in the records are stored (see Figure 2); and annotated sequence *features*, for which feature keys, qualifier keys and values and feature locations are stored. Though output of features into General Feature Format Version 2 (GFF2) [35] is supported, the **Feature **table, one will note, does not explicitly contain source or type fields. This information is stored in other tables and can be pulled together dynamically as a GFF2 record is being constructed. For example, the **BioID_type **table contains the database source information in its db_source field and the internal Atlas **Ontology **table's term field which represents the feature type used in the GFF2 output. However, to reflect the fact that features in such output are now reconstructed from the Atlas system, we prefix the original source type with 'Atlas:', such as in 'Atlas:GenBank/RefSeq'. The reader will note that there are two different Ontology tables in Atlas. A more detailed explanation for the motivation for having two different kinds of Ontology tables is described in the previous Ontology section. However, in the context of sequence features, it is the internal Atlas **Ontology **table that is relevant.

The sequence string is stored in the **Sequence **table. Additional fields for: sequencing technique, tech, such as expressed sequence tags (ESTs); molecule type, moltype, such as DNA, RNA, protein, and nucleic acid; sequence length, length; the NCBI taxonomy identifier, taxonid; and the definition line, defline, are also stored in the Sequence table. Fields such as taxonid, tech, and moltype can be used separately, or in combination to produce customizable queries that return highly specific sets of data. Sequence identifiers, as with all other external identifiers, are managed through a layer of abstraction by associating them with internal identifiers within Atlas, which act as primary keys. Having a single internal identifier for a sequence allows us to relate all other identifiers found in the record to each other. In addition, ontologies for all types of identifiers currently found in GenBank ASN.1 data files, as well as relationships between these identifiers are modeled in the **Bioid **and **Bioid_Bioid **tables, respectively. As mentioned above, sequence features are also modeled in Atlas. For details please refer to the Ontology section below.

#### Molecular interactions

For molecular interaction data, we developed a relational model compliant with the PSI-MI. Adopting a common interaction data model allowed us to unify data from different sources, and allows us to develop a set of common interaction retrieval APIs.

Currently, HPRD, BIND, DIP, IntAct and MINT are included as interaction data sources. BIND, DIP, MINT and IntAct release their data in PSI-MI format. HPRD is releasing data in both PSI-MI standard format, and their own XML format. At the time of this publication, BIND released data as indexed flat files, ASN.1, XML, and PSI-MI format (level 2).

The Atlas interaction model consists of four major entities: **Interactor, Interaction, Experiments **and **Dbxref. Interactor **holds information about one of the interacting members in an interaction, such as the interactor's name, taxonomy, sequence, molecular type, features, subcellular localizations, and external identifiers. An **Interaction **consists of one or more interactors, and one or more experiments.

**Experiment **stores information about the experiments used to identify interactions. Finally, **Dbxref **is used to crosslink the external identifiers such PubMed id, RefSeq accession, HPRD id, BIND id, and Ontology id, for example (see Figure 2). As an additional note, the **Feature **table in the Interaction database is mainly used to store protein features involved in the interactions.

We will release a PSI-MI level 2 compliant version of the Interaction model, and API upon public release of the level 2 specification.

#### Gene related resources

We integrated OMIM, LocusLink, Entrez Gene, HomoloGene and the annotation part of GO into the Atlas system in order to provide gene-related information. Conveniently, the OMIM and LocusLink data sources provide flat file tables which could be imported directly with the MySQL import function. Entrez Gene will eventually replace LocusLink, however in order to maintain a smooth transition and backward compatibility, we are maintaining populated relational models for both Entrez Gene and LocusLink until LocusLink is officially retired. Integration between HomoloGene and Sequence is achieved by relating the taxonomic, protein sequence and gene identifiers with Atlas' **Bioid **table. This allows us to integrate these databases and provide linkage between, for example, orthologous genes present in different interaction scenarios (see Utility of the Atlas system).

### Application programming interfaces

There are two classes of APIs in Atlas: loader and retrieval. Components of Atlas for which we have developed our own relational models, such as the Biological Sequences component, or the Molecular Interactions component, each have their own set of loader APIs. The loader APIs used to build the loading applications, populate instances of the relational models in the Atlas databases. Though most end-users will never need to use the loader APIs, they are critical to the implementation of the Atlas loading process, and are provided to the software development community. The other class of APIs are the retrieval APIs. These APIs serve to retrieve the data stored in Atlas. They are required for developing custom retrieval applications such as the Atlas toolbox applications. The loader API for Biological Sequences is implemented in C++ as it relies heavily on the NCBI C++ Toolkit [36] to parse the ASN.1 data. The Biological Sequence retrieval API, on the other hand, is provided in all three languages: C++, Java, and Perl. The Java and Perl APIs return sequences as BioJava SimpleSequence and BioPerl Bio::Seq objects, respectively. The loader and retrieval APIs for Molecular Interactions are provided in Java. Though retrieval APIs are not supported in all languages, further development in Perl and C++ will be added if our user community requests them. Please refer to Figure 1 for a mapping of data modules to currently supported programming languages. The project is also open source and other developers are encouraged to contribute. All the transactions between the APIs and the database are specified by the numerous SQL statements which are all defined within the majority of the API methods.

#### Application programming interface architecture

**Figure 3:**
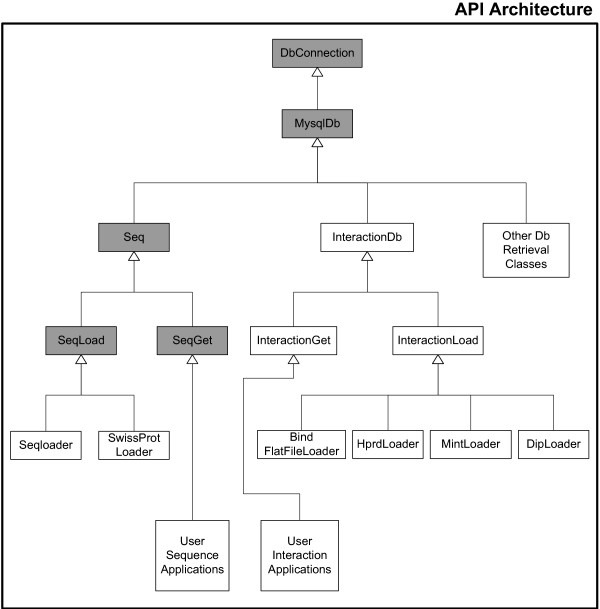
**Atlas API architecture**. MySqlDb, Seq, SeqGet and SeqLoad classes/modules (grey) are available in all three languages: C++, Java, and Perl. The SeqLoad and Seqloader modules are created in C++ only as these are tightly coupled to the NCBI C++ Toolkit. All other classes are available in Java. Applications share the common modules SeqLoad, SeqGet, InteractionLoad, and InteractionGet which provide the methods necessary for loading and retrieval operations, to and from the databases. These modules employ additional classes (not shown) that are representative of the major data model components such as Sequence, Interaction, Interactor, and Dbxref, for example.

The API is constructed using object-oriented methodologies, employing objects to represent everything from low-level database connections to high-level data structures, and their access methods. This is illustrated in Figure 3.

Common in the design of the C++, Java, and Perl APIs, are a set of APIs written for MySQL database connectivity which handles the opening and closing of MySQL connections, as well as managing the execution of the SQL statements themselves. All subsequent APIs that interact with the Atlas database extend from this set of APIs.

Both the data loader and the retrieval utilities share a common class responsible for low-level data transformations. This class includes methods that facilitate conversions between two internal Atlas identifiers, such as bioid_id to ontology_id, or methods that convert internal Atlas identifiers to externally referenced public identifiers, such as GenBank accession numbers, or GI numbers. Inheriting this shared identifier conversion class benefits both the loader APIs and the retrieval APIs, by providing them with the necessary tools to integrate information.

The Biological Sequences component of Atlas manages common identifiers, and hash maps in the **Seq **class. This class is inherited by both the **SeqLoad **class and **SeqGet **class, which define the loader methods, and retrieval methods, respectively. Another feature of the Biological Sequences API, is its ability to control stream output based on molecule types. API users simply specify which molecule type to filter by, through calls to higher-level retrieval methods, and **SeqGet **will then handle the logistics of stream management. Similarly with the Molecular Interactions component of Atlas, the **InteractionDb **class is inherited by the **InteractionLoad **class and the **InteractionGet **class, respectively defining the loader and retrieval methods which manipulate the data in memory.

Our Java interaction APIs, for example, are tightly coupled to our interaction data model with classes representing all the major schema objects such as Interaction, Feature, Dbxref, and Experiment. The APIs are works in progress and we continue to develop and improve them. We are considering even more tightly coupled API development by using XML schema code generators such as JAXB.

All the source code is provided under the GNU General Public License (GPL), and therefore any developer can model future API development on numerous functions we have already implemented.

### Applications

#### Toolbox

The Atlas toolbox is a collection of applications that use the C++ API to perform common sequence and feature retrieval tasks. The applications are standard Unix command-line based tools that follow a command-line option-based interface for parameter entry. These are end-user applications and do not require any programming ability to use them. We have developed toolbox applications for sequence retrieval from accession and GI numbers, retrieval of sequences from all organisms beneath a given node in the NCBI taxonomy tree, retrieval of features given accession and GI numbers, retrieval of sub-sequences corresponding to specific features identified by qualifiers and their values, and retrieval of a set of interactions associated with a molecule given the accession number of an Interactor. Besides being useful tools, the toolbox applications' source code provides good examples of application development using the APIs. Software developers wishing to use the APIs can use these toolbox applications as a starting point for their own custom applications (see Table 2).

#### Data loaders

Data loaders are provided in Atlas to facilitate the parsing and loading of the source datasets into their respective Atlas database tables. Two main classes of loaders are currently supplied in the Atlas package: sequence loaders and interaction loaders. Though other types of data are loaded into Atlas, their loading is trivial as MySQL database dumps of these datasets are already provided by the data providers.

The first class of loaders is the sequence-based loaders. Within this class there are two applications provided: seqloader and fastaloader. The seqloader performs the majority of the sequence loading from GenBank and RefSeq datasets. These datasets have long been represented as ASN.1 (binary/text) by the NCBI [37], and are compact and well defined for storing of structured data. The seqloader was built using the NCBI C++ Software Development Toolkit [36] which was designed to specifically parse the ASN.1 sequence data, extracting such things as the sequence, associated identifiers, features of the sequence and related publications. There are, however, instances where sequence data is missing from the ASN.1 records. In these situations, we obtain the missing records from the NCBI Entrez system in the form of Fasta records. The fastaloader application is then used to update the sequence field in Atlas with the sequences from the Fasta records.

The second class of loaders is interaction-based loaders. These loaders are exclusively implemented in Java. The datasets loaded by this class of loaders include BIND, HPRD, MINT, IntAct and DIP. All the interaction loaders are designed to parse the data in the way that best deals with that particular source data's structure and content (mostly XML). The interaction data is loaded using a common interaction object model, and the interaction loading APIs provide a flexible and extensible framework for future interaction data loading efforts. Currently, we are developing a PSI-MI level 2 data loader.

Besides these classes of loaders, there is also a Java based loader that parses and loads UniProt sequence data. In addition, scripts are used to load datasets for which MySQL dumps, or tab-delimited database dumps are provided. This is handled using the MySQL import function, and eliminates the need to devise special parsers and loaders.

GenBank and RefSeq are checked daily for incremental updates from the NCBI. Accession numbers are used to maintain the integrity of the data. New accession numbers reflect new records and will be inserted into the database. Updated sequences or records with same root accession number and patched annotations will replace existing records in the database. When new releases of GenBank/RefSeq are made available, all databases are purged and reloaded to remove retired records and to maintain referential integrity.

#### Web tools

Though we encourage the use of Atlas as an in-house repository, it can also act to serve the wider internet community. We provide a publicly available web interface to the Atlas databases to demonstrate some of its functionality. This offers basic access to GenBank, RefSeq, NCBI Taxonomy, Atlas Ontologies, BIND, HPRD, MINT, IntAct and DIP. Web interfaces to the Atlas toolbox applications: ac2gi, ac2seq, ac2tax, feat2seq, gi2ac, gi2feat, gi2seq, gi2tax, tax2seq, techtax2seq, tech2seq are available. In addition, interacting partners for proteins identified by accession numbers or GI numbers can be retrieved from any of the four interaction databases stored in Atlas. These web tools can be found at: .

## Utility of the Atlas system

The Atlas data warehouse offers maximum flexibility of data retrieval and integration. Users can access data in Atlas at the SQL, API and end-user application levels. Routine, pre-defined queries can be accessed through the APIs in Java, C++, and PERL (see API section, above), enabling developers to incorporate these queries in their software applications. Most of these queries have been used to build the Atlas toolbox, a set of end-user applications that run on the Unix command-line (Table 2). Included in the toolbox are common utilities for converting GenBank ASN.1 sequences to file formats supported by the NCBI Toolkit [1] such as XML, GenBank Flat File, and FASTA. In addition, information regarding features that are annotated on sequence records can be exported as General Feature Format Version 2 (GFF2). The recently developed General Feature Format Version 3 (GFF3) is not currently supported in Atlas, to allow its specification time to stabilize. However, its support in Atlas is planned in future releases. In the following sections, we illustrate use-cases of the system at the SQL, API and toolbox levels with specific biological themes in mind.

### Single record queries

Single record queries are the simplest use case of the system. Users can input a GenBank or RefSeq accession number and/or GI number into the ac2seq and gi2seq toolbox applications to retrieve the relevant sequence record in Fasta, GenBank or ASN.1 format. Features on a particular sequence can also be retrieved independently with GenBank or RefSeq accession numbers and/or GI numbers. The single record queries can also be performed in batch mode where the user supplies a list of accession numbers or GI numbers and all data pertinent to the list of identifiers is then retrieved.

### Genome annotation

**Figure 4:**
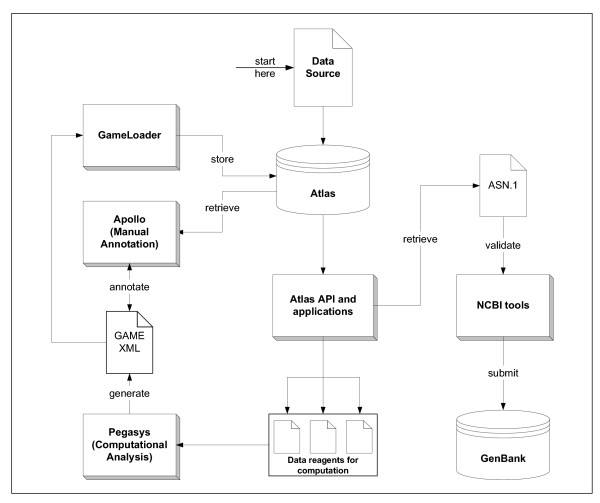
**Using Atlas in genome annotation. **Atlas facilitates genome annotation at multiple levels: creation of data reagents, storage of annotations, and data transformation for submission. Here we show a schema of our genome annotation process that integrates Pegasys, Apollo, NCBI tools and Atlas into a comprehensive platform. Data reagents for sequence alignment are compiled using the Atlas toolbox applications. Computational analyses are run through the Pegasys system which outputs GAME XML for import into Apollo. Annotations are saved in a GAME XML which are then imported into Atlas using the GameLoader. At this step, the biological features created in the annotation process are stored in the Atlas **Feature **tables, exactly the same way a GenBank sequence record containing annotations are stored. These annotations can then be retrieved using the Atlas toolbox application ac2feat and exported in GFF2 or Sequin Feature Table Format for import into the NCBI submission tools for validation, and submission to GenBank.

Atlas provides tools to generate data reagents for genome analysis as well as a data model for storing biological features that have been annotated on the sequences. Coupled with Pegasys [38] and Apollo [39], the Atlas system is an essential part of our annotation platform (see Figure 4). Atlas functions simultaneously as a data reagent generator for sequence alignment analysis, a storage system for annotations that are to be submitted, and a data transformation tool that can convert Apollo-compatible data to NCBI submission tool compatible data.

Atlas provides users with the ability to generate custom sets of data to use as reagents. For example, using tax2seq, users can input a specific node of the NCBI taxonomy tree using its scientific name, or its NCBI taxonomy id and retrieve all nucleotide and amino acid sequences from organisms in the tree rooted at that node. This has special utility in genome analysis where specific sets of data from close relatives of the genome of interest enable comparative genomic methods for functional annotation. Furthermore, this type of taxonomy querying can be combined with the 'tech' field in the NCBI data model to produce sequences derived from different sequencing techniques such as expressed sequence tags (EST), genome survey sequence (GSS), sequence tagged sites (STS), high throughput genomic (HTG), etc. Compiling these specific data sets allows the user to perform more directed sequence similarity searches, for example, that yield more specific hits.

Using the sequence data structure to model existing annotations in sequence records, Atlas can be used to store additional annotations created in Sequin [40] and Apollo [39]. We have built a GAME XML [41] loader that stores annotations exported from Apollo. When used for this purpose, Atlas serves as a holding bay for sequences that can be submitted to DDBJ, EMBL, or GenBank in a relational form that can be mined in the interim using the multi-level query system provided by the Atlas APIs (see Figure 4). Additionally, the annotations stored from a GAME XML [41] file are exportable in GFF2, or Sequin Feature Table Format [42] for use with NCBI submission tools like tbl2asn [42].

### Inference of protein-protein interactions

Deriving new associations from the information extracted from Atlas has proven to be particularly useful in developing a prototype system that infers interactions across species, detailed in "Ulysses – an Application for the Projection of Molecular Interactions across Species" (Kemmer D: in preparation, from the Wasserman and Ouellette laboratories).

Given that the data for protein-protein interactions found within model organisms can be extremely sparse, Ulysses employs homology information to help bridge the gaps in the interaction data by projecting known interactions in one species onto other species for which those interactions are not known, and subsequently inferring potentially novel interactions in those species. Ulysses is able to perform its analyses and inferences, in part, by capitalizing on the integration, offered by Atlas, of HPRD, BIND, and HomoloGene. Atlas makes it possible to retrieve interactions for one species known to occur in another species, by integrating these datasets under one query space, and by providing the API and tools which make such queries simple.

As an example, in both the MINT and DIP databases, protein C-C chemokine receptor type 3 (SwissProt accession number P51677) was found to interact with protein Small inducible cytokine A24 precursor (SwissProt accession number O00175) in human (MINT interaction 14962; DIP interaction 10472E). Although referenced by different publications ([43], [44]), both interactions are likely to be the same. With corroborating evidence for these seemingly synonymous interactions, it can be claimed with more certainty that two proteins do indeed interact. Furthermore, homologs for both sequences can be found in mouse and rat through HomoloGene. Though these homologs are not found to be interacting partners in either mouse or rat, it is reasonable to speculate that such interactions exist in both these organisms.

### Disease-gene associations

The Atlas system is also being used to determine yeast orthologs of genes that are implicated in human disease (Hieter P: in preparation). The inference being that human genes for which there are yeast orthologs represent essential genes which are candidates for human disease agents. Compiling the reagents for this custom database was straightforward using the Atlas tools. It takes advantage of the linkage between sequence identifiers, Taxonomy, HomoloGene, and OMIM.

## Discussion

We have built a data warehouse of biological information with the goal of providing high-throughput, flexible access to the data by means of SQL queries, API-level queries, and end-user application-level queries. Our goal was to create a system that serves as a platform through which information from many sources of data can be interrogated, enabling biologists and computer scientists to easily carry out queries necessary for their research. The data warehouse facilitates complex queries on local instances of GenBank, RefSeq, UniProt, HPRD, BIND, NCBI Taxonomy, HomoloGene, Gene Ontology, OMIM, Entrez Gene, and LocusLink. With previously disparate data now unified in a relational model, SQL can be used to retrieve this consolidated information at once. Though Atlas can act to serve data publicly over the internet, its simple setup enables anyone or any institution to easily serve their own customized data warehouses to their own local users. Installing Atlas in-house to serve local users gives the data provider full control over the data they serve. Giving users access to the system on a high-bandwidth internal network offers convenience and high-performance for large queries, such as retrieving all human ESTs. Such data is then more readily retrieved with lower latency and higher bandwidth than attempting to retrieve the same data over the internet.

One of the important strengths of the Atlas architecture is that it allows data integration at two levels. The first level uses a common data model to integrate similar types of data from different sources (e.g., GenBank or UniProt, and BIND or HPRD). The second level uses the APIs, ontologies, and tools to cross-reference disparate types of data.

For example, consider the task of retrieving all amino acid sequences, and from all organisms found within the taxonomic tree rooted at a given taxonomic node (e.g., *vertebrata*), from the RefSeq database. With a single call to the taxonName2Sequences method, the user can accomplish this task. Within these API methods are SQL statements which first retrieve the taxonid from the **Taxonomy **database. Then using a recursive method, the taxon identifiers for all organisms beneath that given taxon node, are returned. All amino acid sequences for each of these taxon identifiers are then retrieved using taxonId2Sequences (see API documentation [45] for more details).

Uniting disparate sources of data is a useful exercise that highlights the challenges that the data itself presents. Any changes to the source data structure often requires software code changes in order to properly parse the new data format. Failure to do so often leads to the inability to load at least some of the information, if not all. Furthermore, the quality of the original data may often be imperfect as much of this data is curated manually, and hence is subject to data entry errors. Everything from missing data to improperly spelled key terms can impede the loading process. For this reason, it is essential to devise a system that is robust enough to handle unforeseeable exceptions. Policies on how to handle such exceptions are important to define and implement. We try to adhere to the careful logging of incorrect entries that we find during the loading process, and to promptly report these to the data providers for remediation. This is especially important when the specifications of the data are already strictly defined, yet are not followed, or are being misinterpreted.

Semantic inconsistencies may arise due to differences in the interpretation of biological concepts and data, and differences in how such information is mapped into an integration system. That is to say, two systems may contain different data for the same semantic entity. For example, two interaction databases containing localization data for the proteins stored within, may indicate conflicting localization information for a given protein if the set of experimental evidences, used to determine localization, were different between the two systems. Such conflicts between data source providers pose challenges during the integration process as decisions need to be made to resolve the conflict. We continue to evaluate methods of resolving such conflicts. One simple solution is to store the information from all sources as is, and also annotate that information with the source from which it came, so as not to have any information loss. In this way, users can decide on which source they believe and poll the data accordingly. Another solution, which is not as clear cut, would be to selectively merge data, pruning those facts we determine to be incorrect (perhaps based on some measure of consensus between multiple systems), thus leaving only one instance of a factoid in our database. However, as it would not necessarily be our goal to judge the correctness of data, this is perhaps a task better left to users of our system.

### Comparison with other systems

Several other systems are available which have similar goals and provide good solutions to the problem of data integration. We have chosen to discuss Atlas in the context of three other systems: Entrez [11], SeqHound [12] and EnsMart [13]. The Entrez system, produced by the NCBI, provides "an integrated database retrieval system that enables text searching, using simple Boolean queries, of a diverse set of 20 databases". This web-based system is extremely extensive in the scope of data it provides, and in fact many of the Atlas data sources originate from NCBI (GenBank, RefSeq, HomoloGene, Taxonomy, OMIM, Entrez Gene, and LocusLink). The Entrez resources can be found on the NCBI website [46]. In contrast to Entrez, Atlas warehouses the data locally, obviating the need for low-throughput, internet-based queries. Also, additional data sets like HPRD, DIP, MINT and BIND, not currently available through the Entrez interface, have been added to Atlas.

SeqHound [12] is a database of biological sequences and structures, developed by the Blueprint Initiative [47]. SeqHound also stores information on OMIM, LocusLink, and Gene Ontology. SeqHound and Atlas warehouse similar data types. SeqHound provides some different data than Atlas (most notably MMDB). For interaction data, SeqHound utilizes the BIND database. In contrast, Atlas stores interaction data from a number of sources including BIND, HPRD, MINT, DIP, and IntAct. Atlas then, is a more comprehensive repository of interaction data. The major difference between SeqHound and Atlas is in their architectural design. SeqHound stores full records and indexes specific fields which are extracted upon loading. In contrast, Atlas provides relational models for all data sources. This allows SQL-level access to specific parts of the data model. The data in the Atlas relational models are stored as primitive data types as opposed to storing whole records that need parsing or processing. For example, sequences and their annotated biological features can be stored in their own fields in the database, permitting 'substring' operations to extract parts of the sequence that span a particular feature type using SQL. Other systems like EnsMart [13] and the UCSC genome browser [48] have also adopted fully relational models. These systems also provide SQL access over the full data model, and allow arbitrarily complex queries similar to Atlas.

EnsMart is a software system designed by EMBL-EBI [49] and the Sanger Institute [50] which produces and manages automated annotations. The focus of EnsMart is slightly different than Atlas in that its 'core' data is fully sequenced eukaryotic genomes. While information on these genomes is extremely rich in EnsMart and well-integrated using relational models, Atlas attempts to provide a much more extensive source of sequence information. This enables researchers interested in bacteria, viruses, plants or humans to access the system and sources of integrated data with equal facility.

The Atlas system is designed to be locally installed and is not a data provider *per se*, but rather an engine that should be accessed 'in-house'. As with any locally-installable system of this nature, significant time and hardware resources are needed to make the system functional. The utility of the Atlas system will far outweigh the setup time required to get it up and running. Currently, API access to Atlas is limited to the users at the UBC Bioinformatics Centre, University of British Columbia, however the web tools are available worldwide.

### Future work

When working with sources of data from different data providers (for example UniProt and RefSeq), it is advantageous to create mappings from one data source to the other to prevent redundancy and to make associations between proteins to map annotations from one source to the other. We are investigating the idea of an identifier consolidation that can resolve mRNAs and proteins from different sources that are referring to the same protein product to a single identifier.

We will constantly monitor and adjust any change of the data sources. In the near future, we will provide support for a PSI-MI level 2 release, and complete the migration of LocusLink to Entrez Gene. In addition, we are expanding Atlas to include other sources of data. We are currently adding MEDLINE, dbSNP and pathway data to support an integrative genomics and clinical informatics initiative, currently underway in our laboratory. With Atlas in hand, we are also working on an integration project that superimposes co-expression networks derived from microarray experiments and protein-protein interaction networks, to estimate the utility of co-expression networks in inferring protein interactions.

## Conclusion

Atlas is a data warehouse that enables high-throughput, flexible and complex queries on biological data. The system integrates sequences, molecular interactions, taxonomy and homology, and functional annotations on genes. The system functions as data infrastructure to support bioinformatics research and development. Atlas is currently being used in genome annotation projects, disease-gene association projects and inference of molecular interactions. We are releasing Atlas to the scientific community in the hope that it will foster creative ideas for how to make novel associations between disparate sources of data using existing public data sets.

## Availability and requirements

Atlas is available from the UBC Bioinformatics Centre, University of British Columbia. The Atlas package can be downloaded from the Atlas website at: 

The Atlas package contains the Atlas source code and represents the core of the project. The package is distributed under the GNU General Public License. Atlas is designed to run on Unix based systems. Please consult the user manual (available with the distribution) for detailed configuration, compilation and installation instructions. Additional packages are also provided at the website listed above. These packages include a snapshot of the NCBI C++ Toolkit (CVS version 20040505), a MySQL dump of sample data, and additional documentation. The NCBI C++ Toolkit, that is provided, is required only for those users who wish to build the loader applications or for those that require the utilities that convert ASN.1 format to GBFF, EMBL, and XML formats, etc. Those setting up the database will need to install MySQL Server 4.x. Atlas has been tested, specifically, with MySQL Server versions 4.0.9, 4.0.18 and 4.0.20, running on either Linux or AIX.

The Atlas sequence-related binaries (toolbox applications and loader applications) are developed in C++ and therefore a C++ compatible compiler, such as the one included with the GNU GCC suite of tools, should be installed before attempting to build these binaries. We have tested the build process with GNU GCC versions 2.95.3, 2.96, 3.1 and 3.2. In addition, MySQL Client version 4.x and particularly its runtime library, libmysqlclient.a(so), is required. MySQL Client versions 4.0.14 and 4.1.0-alpha were tested. Details on the configuration and use of this library are outlined, in more detail, in the Atlas manual.

For users that require Atlas tools that are based on Java, such as the loading and retrieval tools for LocusLink, BIND, HPRD, and HomoloGene datasets, a compatible Java interpreter must be installed. The API has been tested with J2SE 1.4.1 and J2SE 1.4.2. The Atlas Java API also requires BioJava version 1.4pre, or higher.

For those using the Perl based Atlas tools, a compatible Perl interpreter must be installed. BioPerl version 1.4 must also be installed. Perl version 5.6.1 has been tested.

Each of the packages have their own minimum system requirements. Specific memory, hard disk space and CPU requirements for each package are listed in the manual. As a general guideline, it is essential to have a generous amount of available memory, especially if one anticipates processing large sequences in memory. Another important factor is the amount of available hard disk space. The amount of sequence data to be loaded into Atlas will largely determine your disk space requirements. The Atlas database requires a minimum of 50 GB (RefSeq), plus adequate space for satellite databases. The satellite databases include such things as GO, LocusLink, HPRD, BIND, MINT, and DIP, which are relatively smaller datasets. Note that sequence data can greatly exceed these minimum estimates and the requirements should be carefully planned.

## Authors' contributions

SS was the architect of the system, developed the C++ APIs and wrote the first draft of this manuscript. YH was the database administrator responsible for schema design, data integrity and maintenance. TX contributed the Java APIs. MMSY contributed the PERL APIs. JL developed the C++ APIs, the toolbox and the user manual. BFFO was the principal investigator, conceived of the project and guided its development. JL, YH, MSSY and BFFO all contributed to the writing of this manuscript.

## Figures and Tables

**Table 1:** Data sources included in Atlas.

Atlas Data Source Summary Table *				
**Data Source**	**URL**	**Data Format**	**Atlas Update**	**Update Mechanism**

GenBank Sequence		ASN.1	Daily	Incremental
GenBank Sequence		ASN.1	Release	Reload
GenBank Refseq		ASN.1	Daily	Incremental
GenBank Refseq		ASN.1	Release	Reload
NCBI Taxonomy		Delimited Text	Release	Reload
HomoloGene		Delimited Text	Daily	Reload
OMIM		Delimited Text	Daily	Reload
Gene		Delimited Text	Daily	Reload
LocusLink		Delimited Text	Daily	Reload
UniProt		XML	Bi-weekly	Reload
HPRD		XML	Release	Reload
MINT		XML	Release	Reload
DIP		XML	Release	Reload
BIND		Delimited Text	Release	Reload
GO		MySQL dump	Release	Reload

* up-to-date information about data sources and statistics are available from the Atlas website

**Table 2:** Atlas toolbox applications.

**Application**	**Function**	**Input**	**Output**
			
Sequence			
ac2seq	Retrieve sequences given an accession	Nucleic acid or Protein Accession Number(s)	Sequences in Fasta format
feat2seq	Retrieve sub-sequences that span features	Feature type (and qualifier)	Sequences in Fasta format
gi2seq	Retrieve sequences given a GenInfo identifier	GenInfo Identifier(s) (GI Number(s))	Sequences in Fasta format
gi2seqentry	Retrieve sequences given a GenInfo identifier	GenInfo Identifier(s) (GI Number(s))	GBFF, EMBL, GFF, FTABLE, ASN.1, GBSEQ
tax2seq	Retrieve sequences by taxonomy	NCBI taxon identifier or scientific name of taxon	Sequences in Fasta format
tech2seq	Retrieve sequences by sequencing technique	Sequencing technique (eg EST, GSS, etc.)	Sequences in Fasta format
techtax2seq	Retrieve sequences by taxonomy and sequencing technique	Sequencing technique and NCBI taxonid/scientific name of taxon	Sequences in Fasta format
			

Loader			

fastaloader	Fasta sequence data loader	Sequences in Fasta format	
seqloader	ASN.1 sequence data loader	GenBank/RefSeq ASN.1 records	
			

Feature			

ac2feat	Retrieve features	GenBank Accession number (s)	Features in GFF or FTABLE format
gi2feat	Retrieve features	GenInfo Identifier(s) (GI Number(s))	Features in GFF or FTABLE format
			

Taxonomy			

ac2tax	Retrieve taxonomy given an accession number	GenBank Accession number (string)	NCBI taxon identifier (integer)
gi2tax	Retrieve taxonomy given a GenInfo identifier	GenInfo Identifier (integer)	NCBI taxon identifier (integer)
			

ID Converters			

ac2gi	Convert an accession number to a GenInfo identifier	GenBank Accession number (string)	GenInfo Identifier (integer)
gi2ac	Convert a GenInfo identifier to an accession number	GenInfo Identifier (integer)	Accession number (string)
tax2gi	Retrieve GenInfo identifiers associated with taxon identifier	NCBI taxon identifier (integer)	GenInfo Identifier (integer)
